# Clinical Experience with Frame Based Stereotactic Biopsy for Intracranial Space Occupying Lesion

**DOI:** 10.31729/jnma.3665

**Published:** 2018-08-31

**Authors:** Suresh Bishokarma, Shikher Shrestha, Munu Napit, Dinesh Nath Gongal

**Affiliations:** 1 Department of Neurosurgery, Upendra Devkota Memorial National Institute of Neurological and Allied Sciences, Bansbari, Kathmandu, Nepal

**Keywords:** *biopsy*, *brain tumors*, *computed tomography*, *stereotaxy*

## Abstract

**Introduction:**

Preoperative diagnosis of intracranial space occupying lesion based solely on clinical and neuroimaging evaluation may not be sufficient to institute treatment plan without histopathological certainty. Frame based stereotactic biopsy is a technique of retrieving biopsy specimen to determine the histopathology. The aim of this study is to assess the efficacy and accuracy of frame based technique.

**Methods:**

This is a cross-sectional study conducted among 80 patients who underwent computed tomography guided frame based stereotactic biopsy during a period of 6 years. All operations were performed under local anesthesia. Histopathology reports were retrieved and accuracy of biopsy technique analyzed.

**Results:**

Out of 80 patients, 58 were male with male to female ratio of 2.6:1. Median age of patients were 50 years with range from 16 to 75 years. Most lesions were in deeper location 49 (61.3%). Most common location was Parietal, 15 (18.8%) followed by Thalamic, 12 (15%). Mean size of lesion was 2.88±0.71cms ranged from 2 to 5cms. Biopsy was accurate to retrieve target in 74 (92.5%) patients. Histopathology revealed glial tumor in 41 (51.2%) of cases. Overall morbidity was observed in 3 (5.5%) patients. There is no procedure related mortality in this study during study period.

**Conclusions:**

Frame based biopsy of intracranial space occupying lesion is safe and efficacious procedure with high diagnostic yield.

## INTRODUCTION

Preoperative diagnosis of intracranial space occupying lesion based solely on clinical and neuroimaging evaluation may not be sufficient to institute treatment plan without histopathological certainty. Histopathological diagnosis is always necessary to make an effective treatment plan for intracranial mass lesions.

Computed Tomography (CT) guided frame based stereotactic biopsy is a minimally invasive procedure that uses three dimensional (3D) coordinated system for precisely locating lesion to obtain tissue sample for histopathological examination. It is an extremely safe and effective procedure for determining the histopathological diagnosis of intracranial lesions.^1^

The aim of this study is to assess the efficacy and accuracy of frame based stereotactic biopsy technique.

## METHOD

This is a descriptive cross-sectional study conducted in Upendra Devkota Memorial National Institute of Neurological and Allied Sciences (UPMNINAS), Kathmandu, Nepal among 80 patients, who underwent frame based stereotactic biopsy during six years period from March 2012 to Jan 2017. All operations were performed under local anesthesia. Ethical approval was taken from UDMNINAS, IRC. Deeper lesion and those patients with superficial location who cannot withstand major surgical procedure were included. Superficial lesion with good KPS score who underwent direct surgical excision were excluded. Convenience (non-probability) sampling method was used and sample size of 80 was calculated using following formula.


$$\text{Sample size }=\text{ (Z}\times {\text{P(1-P)/e}}^{\text{2}}\text{) (1 }{\text{+Z}}^{2}\times \text{P}\left(1-\text{P}\right)/{\text{e}}^{2}\text{N)=78.6}$$

Where Population size (N)=700; Confidence level (%) = 95; P = 0.333 with Margin of error (e) = 0.04.

Statistical package for social science (SPSS) version 20.0 was used for the data entry and analysis. The data were presented and the outcomes were analyzed using Chi-square test. Proportion and mean were deduced for categorical data and continuous variables respectively. P<0.05 was considered significant.

Technique of frame-based biopsy: Preoperatively patients were assessed clinically and available records such as CT scan, chest x-ray evaluated. Coagulation profile is checked. Head of the patient was shaved or washed with antiseptics and base ring of Brown-Roberts-Wells (BRW) system was secured in to the outer table of the skull with four screws after infiltrating the required points with 2% lignocaine. Patient was shifted to CT scanner. Localizing ring was attached to base ring before CT scanning. Contrast enhanced computed tomography was done in each patient. Areas with contrast enhancement was selected while areas of most suspicion were selected for non-enhancing lesion. Pixel coordinates of nine localizer rods were derived and recorded. X and Y coordinates was calculated with Radionic Stereocalc application in Windows Microsoft software and three scales (Antero-posterior, lateral and vertical) were calculated. Patient shifted to OT. Calculation were calibrated to phantom target. Patient head is prepped and draped. Entry point was infiltrated with 2% lignocaine, incised and small burr hole made with Hudson or Manman perforator. Durotomy was made with electrocautery. Cosman-Roberts-Wells (CRW) frame was mounted on the head. A side cutting biopsy needle was used and an average of 4 specimens were obtained through single trajectory and sent for histopathological analysis. Wound closed with one or two stitches and base ring removed and patient observed in post-operative unit.

## RESULTS

Out of 80 patients who underwent frame based biopsy of intracranial space occupying lesion, most of them were male (58 patients) with male to female ratio of 2.6:1. Median age of patients was 50 years with range from 16 to 75 years. Most lesions were observed in right side 42 (52.5%) while 38 (47.5%) lesions were in left side.

**Figure 1.**
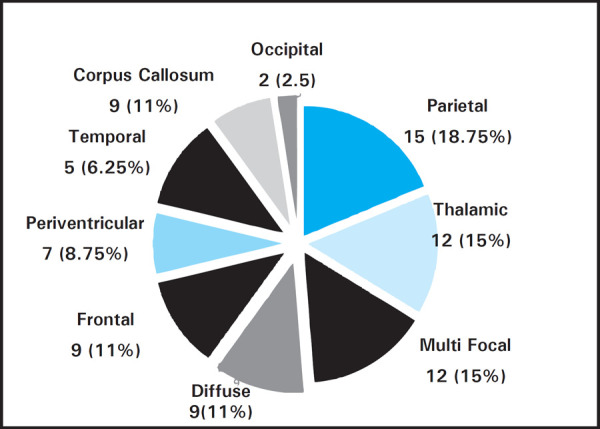
Location of intracranial space occupying lesion.

Deep location was found in 49 (61.25%) patients while lobar location was found in 31 (38.8%) patients. Most common location was parietal 15 (18.8%) followed by thalamic 12 (15%), multifocal 12 (15%), Frontal 9 (11%), Corpus callosum 9 (11%) periventricular 7 (8.75%) while 9 (11.3%) were diffuse (Figure 1). Mean size of lesion was 2.88±0.71cms ranged from 2 to 5 cms. 56 (70%) patients had lesion size more than 2 cms while 24 (30%) patients had lesion size ≤2cms.

**Figure 2.**
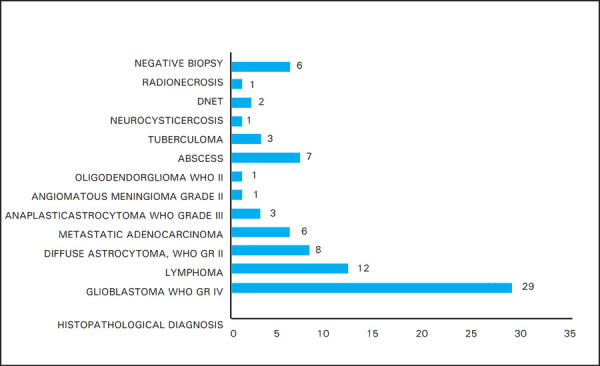
Histopathological diagnosis.

Biopsy was accurate to retrieve target in 74 (92.5%) patients (Table 1). Histopathology revealed glial tumor in 41 (51.2%) of cases. Out of 80 biopsies, 29 were Glioblastoma WHO Grade IV (36.3%), 12 were lymphoma (15%), 8 were diffuse astrocytoma (10%), followed by few cases of abscess, Anaplastic astrocytoma WHO grade III, tuberculoma, cryptococcal lesion, metastatic lesion etc (Figure 2).

**Table 1:** Diagnostic yield of frame based stereotactic biopsy.

Frame-based stereotactic biopsy	n (%)
No. of biopsy	80 (100%)
Positive biopsy	74 (92.5%)
Negative biopsy	6 (7.5%)

HPE was inconclusive in 6 (7.5%) cases. Among 6 inconclusive diagnosis, gliosis was reported in 3 cases, normal brain in 2 patients and a chronic inflammatory neuroparenchyma in HPE report (Table 2). With frame based technique, tissue diagnosis was made in 74 (92.5%). New diagnosis was revealed in 11 (13.6%) patients. While diagnosis was static to preoperative diagnosis in 63 (78.75%) patients (Table 3).

**Table 2:** Inconclusive diagnosis

Negative reports	n (%)
Gliosis	3 (3.75)
Normal brain	2 (2.5)
Chronic inflammatory neuroparenchyma	1 (1.25)
Total	6 (7.5)

Overall morbidity was observed in 3 (5.5%) patients. Two patients developed seizure and one patient had tract hematoma which was managed conservatively (Table 4). There is no procedure related mortality in our study.

**Table 3:** Alteration in preoperative diagnosis following Frame-based stereotactic biopsy.

Change in diagnosis	n (%)
Preoperative diagnosis static	63 (78.75)
Diagnosis revised	11 (13.75)
Inconclusive	6 (7.5)
Total	80 (100)

**Table 4:** Morbidity related to frame based stereotactic biopsy.

Morbidity	n (%)
Seizure	2 (2.5)
Tract hematoma	1 (1.25)
Total	3 (3.75)

## DISCUSSION

With the advent of computed tomography (CT) in the 1970s-precise visualization of the location of lesions affecting the central nervous system (CNS) was possible. CT-guided freehand techniques were used to obtain tissue from intracranial lesions until rigidly fixed stereotactic headframes were developed in the early 1980s.^2^ Literature comparing diagnostic yield of stereotactic technique is sparse. Hence this study was undertaken to evaluate diagnostic yield of frame based stereotactic technique.

The diagnostic accuracy of frame based stereotactic technique were reported from 84.21% to 97.5%^3,4–7^ in different literatures. In our study, diagnostic accuracy of frame-based stereotactic technique was 92.5%. In a study done by Jain D et al.^3^ among 95 patients, conclusive diagnosis was made in 80 patients (84.21%) while Dorward et al.^8^ had diagnostic yield of 94.9% among 75 frame based biopsy.

Mean size of lesions in our study was 2.88±0.71cms ranged from 2 to 5cms. In a study done by Dorward et al. the mean size of lesion was 3.48±1.62cms with range from 0.8cm to 8cms among frame based group.^8^ Various literature suggested the volume of brain lesion influences the diagnostic yield. The larger the lesion, the greater is the likelihood biopsy accuracy and vice versa. However, we didn't find any significant difference in diagnostic yield among lesion ≤2cm or >2cm size (P=0.413). This could be due heterogeneous distribution of small size lesion with predominant larger lesion. The reason could be due to delayed presentation because of poor referral system in a developing country like Nepal.

In our study, histopathology revealed glial tumor in 41 (51.2%) of cases. Out of 80 biopsies, 29 were Glioblastoma WHO Grade IV (36.3%), 12 were Lymphoma (15%), 8 were Diffuse astrocytoma WHO Gr II (10%), 11 being other tumors (13.75%), abscess (8.8%), tuberculoma (3.8%) etc. In a study done by Tsermoulas G et al.^6^ among 124 patients, diagnostic accuracy was 93.5% with Glioblastoma being the most common (41.1%) followed by B cell lymphoma (17.74%) which was comparable to our study.

Among 6 inconclusive reports, 3 were gliosis, 2 were normal brain and a chronic inflammatory neuroparenchyma. Reason for negative report were due to missed target acquiring normal brain for histology or retrieval of glial tissue/nonspecific chronic inflammatory tissue from target. Study done by Jain D et al.^3^ had overall negative result in 15 (15.79%) out of 95 patients. In their study, histology revealed normal brain in 10 (10.5%)out of 95 patients, gliosis in 4.2% cases and inadequate tissue in 1.05% cases. ^3^

The overall morbidity of needle biopsy is reported from 0.9% to 15% in different literature. ^5,9,10^ In our study, overall morbidity was observed in 3 (5.5%) patients. Kreth et al. highlighted hematoma related complication as a common.^10^ One patient in our study (1.25%) had tract hematoma which was managed conservatively. Two patients (2.5%) developed seizure.

## CONCLUSIONS

Frame based biopsy of intracranial space occupying lesion is safe and efficacious procedure with high diagnostic yield. We recommend future prospective study to compared frame based technique with other needle biopsy technique to ascertain and compare its accuracy, efficacy and safety.

## References

[1] Hall WA. (1998). The Safety and Efficacy of Stereotactic Biopsy for Intracranial Lesions. Cancer. Medicine (Baltimore)..

[2] Wen DY, Hall WA, Miller DA, Seljeskog EL, Maxwell RE. (1993). Targeted brain biopsy: a comparison of freehand computed tomography-guided and stereotactic techniques. Neurosurgery..

[3] Jain D, Sharma MC, Sarkar C, Deb P, Gupta D, Mahapatra AK. (2006). Correlation of diagnostic yield of stereotactic brain biopsy with number of biopsy bits and site of the lesion. Brain Tumor Pathol..

[4] Alkhani AM, Ghosheh JM, Al-Otaibi F, Ghomraoui AH, Kanaan IN, Hassounah MI. (2008). Diagnostic yield of stereotactic brain biopsy. Neurosciences (Riyadh)..

[5] Apuzzo ML, Chandrasoma PT, Cohen D, Zee CS, Zelman V. (1987). Computed imaging stereotaxy: experience and perspective related to 500 procedures applied to brain masses. Neurosurgery.

[6] Tsermoulas G, Mukerji N, Borah AJ, Mitchell P, Nicholas Ross N. (2013). Factors affecting diagnostic yield in needle biopsy for brain lesions. British Journal of Neurosurgery..

[7] Joshi RM, Lohani S, Devkota UP. (2015). Computed tomography guided stereotactic biopsy of cerebral lesion: A safe diagnostic procedure. Nepal Journal of Neurosciences..

[8] Dorward NL, Paleologos TS, Alberti O, Thomas DGT (2002). The advantages of frameless stereotactic biopsy over frame-based biopsy. British Journal of Neurosurgery..

[9] Krieger MD, Chandrasoma PT, Zee CS, Apuzzo ML. (2001). Role of stereotactic biopsy in the diagnosis and stereotactic biopsy of intra-axial brain tumors — a prospective study. Acta Neurochir..

[10] Kreth FW, Muacevic A, Medele R, Bise K, Meyer T, Reulen HJ. (2001). The risk of hemorrhage after image guided stereotactic biopsy of intra-axial brain tumors—a prospective study. Acta Neurochir..

